# Hemodynamic Gain Index Is Associated With Cardiovascular Mortality and Improves Risk Prediction: A PROSPECTIVE COHORT STUDY

**DOI:** 10.1097/HCR.0000000000000777

**Published:** 2023-03-06

**Authors:** Jari A. Laukkanen, Nzechukwu M. Isiozor, Peter Willeit, Setor K. Kunutsor

**Affiliations:** Central Finland Health Care District Hospital District, Department of Medicine, Finland District, Jyväskylä, Finland, and Institute of Public Health and Clinical Nutrition, University of Eastern Finland, Kuopio, Finland (Dr Laukkanen); Institute of Clinical Medicine, Department of Medicine, University of Eastern Finland, Kuopio, Finland (Drs Laukkanen and Isiozor); Clinical Epidemiology Team, Medical University of Innsbruck, Innsbruck, Austria, and Department of Public Health and Primary Care, University of Cambridge, Cambridge, United Kingdom (Dr Willeit); and Diabetes Research Centre, University of Leicester, Leicester General Hospital, Leicester, United Kingdom, and National Institute for Health Research Bristol Biomedical Research Centre, University Hospitals Bristol NHS Foundation Trust and University of Bristol, Bristol, United Kingdom, and Musculoskeletal Research Unit, Translational Health Sciences, Bristol Medical School, University of Bristol, Learning & Research Building (Level 1), Southmead Hospital, Bristol, United Kingdom (Dr Kunutsor).

**Keywords:** cardiopulmonary exercise testing, cardiorespiratory fitness, cardiovascular disease, cohort study, exercise hemodynamics, mortality

## Abstract

**Purpose:**

The hemodynamic gain index (HGI) and cardiorespiratory fitness (CRF) are parameters assessed during cardiopulmonary exercise testing (CPX). The association between the HGI and cardiovascular disease (CVD) mortality is uncertain. We evaluated the association between the HGI and CVD mortality risk using a prospective study.

**Methods:**

The HGI was calculated using heart rate (HR) and systolic blood pressure (SBP) measured in 1634 men aged 42-61 yr during CPX, using the formula: [(HR_peak_× SBP_peak_) − (HR_rest_ × SBP_rest_)]/(HR_rest_ × SBP_rest_). Cardiorespiratory fitness was directly measured using a respiratory gas exchange analyzer.

**Results:**

During a median (IQR) follow-up of 28.7 (19.0, 31.4) yr, 439 CVD deaths occurred. The risk of CVD mortality decreased continuously with the increasing HGI (*P* value for nonlinearity = .28). Each unit higher HGI (1.06 bpm/mm Hg) was associated with a decreased risk of CVD mortality (HR = 0.80: 95% CI, 0.71-0.89), which was attenuated after further adjustment for CRF (HR = 0.92: 95% CI, 0.81-1.04). Cardiorespiratory fitness was associated with CVD mortality and the association remained after adjustment for the HGI: (HR = 0.86: 95% CI, 0.80-0.92) per each unit (MET) higher CRF. Addition of the HGI to a CVD mortality risk prediction model improved risk discrimination (C-index change = 0.0285; *P* < .001) and reclassification (net reclassification improvement = 8.34%; *P* < .001). The corresponding values for CRF were a C-index change of 0.0413 (*P* < .001) and a categorical net reclassification improvement of 14.74% (*P* < .001).

**Conclusions:**

The higher HGI is inversely associated with CVD mortality in a graded fashion, but the association is partly dependent on CRF levels. The HGI improves the prediction and reclassification of the risk for CVD mortality.

## Abstract 2

Higher HGI during cardiopulmonary exercise testing is inversely associated with CVD mortality in a graded fashion in 1634 men aged 42-61 years, but the association is partly dependent on CRF levels. HGI improves the prediction and reclassification of the risk for CVD mortality. However, CRF remains a stronger risk predictor of CVD mortality compared to HGI.

**KEY PERSPECTIVES**

- Hemodynamic gain index (HGI) is a novel hemodynamic marker developed from the combination of exercise heart rate and systolic blood pressure responses.
- This is the first population-based study on the association between HGI and the risk of CVD mortality.
- Higher HGI during exercise test was associated with a lower risk of CVD mortality in a graded fashion and improved risk prediction.

Despite many advances in the development of effective cardiovascular disease (CVD) prevention and management strategies, it still remains the leading cause of mortality.^1^ Major risk factors for CVD include hypertension, diabetes, obesity, smoking, and dyslipidemia,^2^ but these do not fully explain the risk for CVD. Thus, the identification of easily measurable risk markers that have better predictive relevance may help stem the public health burden attributed to CVD.

Cardiorespiratory fitness (CRF), which is a comprehensive and integrative measure of whole-body physical function,^3^ is increased through increased aerobic endurance exercise.^3^ It is well established that CRF is strongly, inversely, and independently associated with CVD and mortality outcomes^4^–^9^ and improves risk prediction when added on top of traditional risk factors^10^,^11^; it is a stronger risk predictor of CVD risk compared with many common risk factors.^12^ The American Heart Association has advocated the routine assessment of CRF as a clinical vital sign when assessing other traditional risk factors.^3^ The gold standard for quantifying CRF is peak oxygen uptake (V˙o_2peak_), which is measured during maximal cardiopulmonary exercise testing (CPX).^13^,^14^ Cardiorespiratory fitness and hemodynamic responses such as exercise heart rate (HR) and blood pressure (BP) are core parameters that are assessed during exercise testing, and are commonly used in the evaluation of CVD risk.^13^,^15^,^16^ The hemodynamic gain index (HGI), a novel hemodynamic marker developed from the combination of exercise HR and systolic blood pressure (SBP) responses,^17^ is correlated with CPX parameters such as V˙o_2peak_ and has been shown to be strongly and independently associated with adverse cardiovascular outcomes such as all-cause mortality.^17^–^20^ The association between the HGI and the risk of CVD mortality is uncertain. Using a population-based prospective cohort study comprising middle-aged and older Finnish males, our primary objective was to evaluate the nature and magnitude of the association between the HGI and the risk of CVD mortality. A second primary objective was to investigate the extent to which HGI measurements could improve the risk prediction of CVD mortality when added to conventional risk factors. In subsidiary analyses, we compared the HGI with CRF as risk and prognostic indicators for CVD mortality in the same sample of participants.

## METHODS

**Figure 1.**
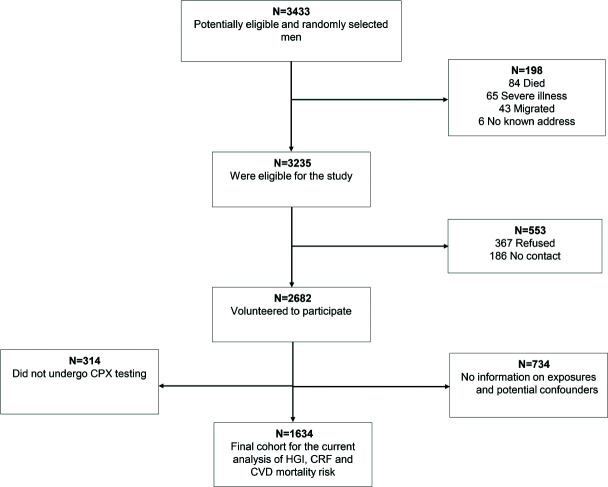
Participant flow through the study. Abbreviations: CPX, cardiopulmonary exercise testing; CVD, cardiovascular disease; HGI, hemodynamic gain index.

The current study was conducted and reported in accordance with STROBE (STrengthening the Reporting of OBservational studies in Epidemiology) guidelines for reporting observational studies in epidemiology (see Supplemental Digital Content 1, available at: http://links.lww.com/JCRP/A453).^21^ We employed data from the Kuopio Ischaemic Heart Disease (KIHD) risk factor study, a general population-based prospective cohort study based in Finland designed to evaluate potential risk factors for atherosclerotic cardiovascular outcomes and other related chronic diseases. Study participants who were recruited comprised a representative sample of men aged 42-61 yr who were residents in the city of Kuopio and its surrounding rural communities in eastern Finland. Invitations were initially sent to potential study participants during the recruitment phase. Of the 3433 potentially eligible men who were invited for baseline screening examinations between March 1984 and December 1989, 3235 were found to be eligible. Of this number, 553 did not respond to the invitation or declined to give informed consent, which left 2682 (83%) men who volunteered to participate in the study.^6^ Of this number, 734 men had missing data on relevant covariates and 314 men did not undergo exercise testing because of musculoskeletal problems. The current analysis is based on 1634 men who had complete data on the HGI, CRF as measured by V˙o_2peak_, relevant covariates, and CVD mortality events (Figure 1). The Research Ethics Committee of the University of Eastern Kuopio, Finland, approved the study, which was conducted in accordance with the Declaration of Helsinki. All study participants provided written informed consent.

### ASSESSMENTS

A maximal symptom-limited cycle exercise-tolerance test was conducted using an electronic braked ergometer between 8:00-10:00 am.^22^ The HGI was estimated using HR and SBP responses from exercise tests using the formula: ([HR_max_ × SBP_max_] − [HR_rest_ × SBP_rest_])/(HR_rest_× SBP_rest_).^17^ Peak oxygen uptake, used as a measure of CRF, was assessed directly using a respiratory gas analyzer (Medical Graphics, MCG). The standardized testing protocol included a 3-min warm-up at 50 W (1 W = 6.12 kg⋅m/min), followed by 20-W/min increases in workload with direct analyses of expired respiratory gases. V˙o_2peak_ was defined as the highest or peak attained value for V˙o_2_ and was expressed as both mL/kg/min and metabolic equivalents (METs; 1 MET corresponding to a V˙o_2_ of 3.5 mL/kg/min). Electrocardiographic indices, BP, and HR were measured at rest and during exercise testing.^23^,^24^ All exercise tests were performed under the supervision of an experienced physician and nurse.

Assessments of sociodemographic characteristics, medical histories, and physical examinations and measurements of blood biomarkers have been described previously.^25^–^28^ Prior to blood sample collection between 08:00-10:00 am, participants were instructed to fast overnight, abstain from drinking alcohol for ≥3 d and from smoking. The collected serum samples were stored frozen at −80°C for 0.2-2.5 yr. The cholesterol content of lipoprotein fractions was measured enzymatically (Boehringer Mannheim). Fasting plasma glucose (FPG) was measured by the glucose dehydrogenase method (Merck) after protein precipitation by trichloroacetic acid. Measurements of serum high-sensitivity C-reactive protein (hsCRP) were made with an immunometric assay (Immulite High Sensitivity C-Reactive Protein Assay; DPC). Self-administered questionnaires were used to assess socioeconomic status (SES), smoking status, alcohol consumption, baseline diseases, and use of medication. Socioeconomic status involved a summary index that combined factors such as income, education, occupational prestige, material standard of living, and housing conditions. The composite SES index ranged from 0-25, with higher values indicating lower SES.^29^–^31^ Smoking status was categorized as smokers and nonsmokers, where a smoker was classified as one who had smoked regularly and had smoked cigarettes, cigars, or a pipe within the past 30 d. The Nordic Alcohol Consumption Inventory was used to assess alcohol consumption, which was reported in g/wk. Body mass index (BMI) was computed as the ratio of weight (kg) to the square of height (m). The energy expenditure of physical activity (PA) was assessed using the validated KIHD 12-mo leisure-time PA questionnaire.^25^

### ASCERTAINMENT OF OUTCOMES

Cardiovascular deaths that occurred from study enrollment to December 31, 2018, were included in the current analysis. All KIHD study participants are under continuous annual surveillance for outcomes including mortality events using personal identification codes.^32^ No losses to follow-up have been recorded in the KIHD study. Cardiovascular deaths were ascertained from death certificates, hospital records, local hospitals, informant interviews, health practitioner questionnaires, study electrocardiograms, medicolegal reports, and vital statistics offices. The outcomes were coded using the *International Classification of Diseases, Ninth Revision (ICD-9)* and *International Statistical Classification of Diseases, 10th Revision (ICD-10),* codes. Documents were cross-checked in detail by two physicians. The Independent Events Committee, masked to clinical data, performed classification of outcomes.

### STATISTICAL ANALYSIS

Descriptive statistics were used to summarize the baseline data: mean ± SD or median (IQR) for continuous variables based on the distribution of the data and n (%) for categorical variables. Cross-sectional associations of the HGI with several risk markers were evaluated by calculating Pearson's correlation coefficients and using linear regression models adjusted for age. The HR with 95% CI for CVD mortality were estimated using Cox proportional hazard regression models, after confirming no statistically significant departure from the assumptions of the proportionality of hazards using Schoenfeld residuals.^33^ To evaluate the potential nonlinear relationship between the HGI and CVD mortality, we constructed a multivariable restricted cubic spline curve with knots at the 5th, 35th, 65th, and 95th percentiles of the distribution of the HGI as recommended by Harrell.^34^ Given evidence of a departure from nonlinearity, the HGI was modeled as a continuous (per unit increase) variable, with subsidiary modeling using tertiles. To assess the independence of the association, HR were progressively adjusted for using three models: (model 1) age; (model 2) model 1 plus smoking status, history of type 2 diabetes (T2D), total cholesterol, high-density lipoprotein cholesterol (HDL-C), BMI, FPG, alcohol consumption, prevalent coronary heart disease (CHD), use of cholesterol medication, prevalent atrial fibrillation (AF), total PA, SES, and hsCRP; and (model 3) model 2 plus CRF. Cardiorespiratory fitness is well established to be strongly associated with CVD mortality and there is a significant correlation between the HGI and CRF.^17^ Hence, we also assessed the association between CRF and CVD mortality in the same set of participants with consistent adjustment for confounders to make direct comparisons with that of the HGI and CVD mortality. To examine whether the HGI and CRF were each associated with CVD mortality independent of each other, we controlled the HGI for CRF and CRF for the HGI (model 3). Covariates selected for adjustment were based on (i) their roles as established risk factors for CVD, (i) evidence from previous research including the KIHD study,^11^,^26^,^27^,^35^,^36^ or (iii) their potential as confounders based on known associations with CVD and observed associations with exposures using the available data. To assess statistical evidence of effect modification on the HGI-CVD mortality association by clinically relevant characteristics, we performed subgroup analyses using tests of interaction.

To assess whether adding information on the HGI to established risk factors is associated with improvement in prediction of CVD mortality risk, we calculated measures of discrimination for censored time-to-event data (Harrell's C-index^37^) and reclassification.^38^,^39^ To investigate the change in C-index on the addition of the HGI, two CVD mortality risk prediction models were fitted: one model based on traditional risk factors (ie, age, history of diabetes, total cholesterol, HDL-C, and smoking) and the second model with these risk factors plus the HGI. The 95% CI for C-indices and their changes were derived from jackknife standard error. In addition to Harrel's C-index, we tested differences in the −2 log likelihood of prediction models with and without inclusion of the HGI. The −2 log likelihood test has been recommended as a more sensitive risk discrimination method.^40^,^41^ Reclassification analyses were restricted to the first 25 yr given the long follow-up of the cohort and were assessed using the net reclassification improvement^38^,^39^ and integrated discrimination improvement^38^ by comparing the model containing conventional risk factors to the predicted risk from the model containing conventional risk factors plus the HGI. Reclassification analyses were based on predicted 25-yr CVD mortality risk categories of low (<8%), intermediate (8-30%), and high (>30%) risk as previously reported.^26^,^27^,^42^ Given that it is well documented that CRF provides additional prognostic values beyond established risk factors in predicting fatal cardiovascular outcomes,^10^,^42^–^44^ we also compared the predictive ability of the HGI with CRF in the same participants. All statistical analyses were conducted using Stata MP version 17 (Stata Corp).

## RESULTS

The age, HGI, and CRF at baseline were 52 ± 5 yr, 2.56 ± 1.06 bpm/mm Hg, and 8.7 ± 2.3 METs, respectively (Table 1). Inverse correlations of weak to moderate strength were observed between the HGI and the following risk markers: resting HR and SBP on the ergometer, age, SES, BMI, BP, total cholesterol, FPG, and hsCRP (Table 1). The hemodynamic index was weakly and positively correlated with PA and HDL-C; correlations with peak HR and SBP on the ergometer and CRF were moderately strong. Values of the HGI were significantly lower in men with T2D compared with men without T2D, smokers compared with nonsmokers, and men with prevalent CHD compared with men without prevalent CHD.

**Table 1:** Baseline Participant Characteristics and Correlates of the Hemodynamic Gain Index^a^

	Mean ± SD, n (%), Median (IQR)	Pearson Correlation *r* (95% CI)^b^	Percentage Difference (95% CI) in Values of HGI per 1 SD Higher or Compared to Reference Category of Correlate^c^
*Exposure and related measures*			
HGI, bpm/mm Hg	2.56 ± 1.06	...	...
Resting heart rate on bicycle, bpm	62.1 ± 10.6	−0.51 (−0.54 to −0.47)^d^	−0.50 (−0.54 to –0.46)^d^
Peak heart rate on bicycle, bpm	155.8 ± 24.7	0.54 (0.51-0.57)^d^	0.59 (0.54-0.63)^d^
Resting SBP on bicycle, mm Hg	150 ± 22	−0.38 (−0.42 to −0.34)^d^	−0.39 (−0.43 to −0.34)^d^
Peak SBP on bicycle, mm Hg	203 ± 27	0.35 (0.31-0.39)^d^	0.35 (0.31-0.40)^d^
CRF, METs	8.72 ± 2.29	0.54 (0.50-0.57)^d^	0.57 (0.53-0.62)^d^
*Questionnaire/prevalent conditions*			
Age at survey, yr	52 ± 5	−0.35 (−0.39 to −0.30)^e^	−0.37 (−0.41 to −0.32)^e^
Alcohol consumption, g/wk	44.0 (12.6, 104.9)	−0.02 (−0.07 to 0.02)	−0.02 (−0.07 to 0.02)
Socioeconomic status	8.13 ± 4.26	−0.18 (−0.23 to −0.13)^d^	−0.18 (−0.23 to −0.13)^d^
History of type 2 diabetes			
No	1548 (94.7)	...	Reference
Yes	86 (5.3)	...	−0.62 (−0.83 to −0.40)^d^
Smoking status			
Other	1069 (65.4)	...	Reference
Current	565 (34.6)	...	−0.26 (−0.36 to −0.16)^d^
History of CHD			
No	1258 (77.0)	...	Reference
Yes	376 (23.0)	...	−0.67 (−0.78 to −0.56)^d^
History of AF			
No	1614 (98.8)		Reference
Yes	20 (1.2)		0.06 (−0.38 to 0.49)
Medication for dyslipidemia			
No	1625 (99.4)	...	Reference
Yes	9 (0.6)	...	−0.29 (−0.94 to 0.36)
*Physical measurements*			
BMI, kg/m^2^	26.9 ± 3.4	−0.18 (−0.23 to −0.14)^d^	−0.18 (−0.23 to −0.14)^d^
SBP, mm Hg	134 ± 16	−0.28 (−0.32 to −0.23)^d^	−0.28 (−0.33 to −0.23)^d^
DBP, mm Hg	89 ± 10	−0.25 (−0.29 to −0.20)^d^	−0.24 (−0.29 to −0.20)^d^
Physical activity, KJ/d	1217 (662, 2006)	0.13 (0.08-0.17)^d^	0.13 (0.08-0.17)^d^
*Blood-based markers*			
Total cholesterol, mmol/L	5.94 ± 1.08	−0.07 (−0.11 to −0.02)^f^	−0.07 (−0.11 to −0.02)^f^
HDL-C, mmol/L	1.28 ± 0.30	0.15 (0.10-0.20)^d^	0.15 (0.10-0.20)^d^
Fasting plasma glucose, mmol/L	5.34 ± 1.22	−0.16 (−0.21 to −0.11)^d^	−0.16 (−0.21 to −0.11)^d^
High-sensitivity CRP, mg/L	1.26 (0.69-2.38)	−0.24 (−0.28 to −0.19)^d^	−0.24 (−0.28 to −0.19)^d^

Abbreviations: AF, atrial fibrillation; BMI, body mass index; CHD, coronary heart disease; CRF, cardiorespiratory fitness; CRP, C-reactive protein; DBP, diastolic blood pressure; HDL-C, high-density lipoprotein cholesterol; HGI, hemodynamic gain index; MET, metabolic equivalents; SBP, systolic blood pressure.

^a^SBP and DBP were measured at rest.

^b^Age-adjusted Pearson correlation coefficients between the HGI and the row variables.

^c^Age-adjusted percentage change in values of the HGI per 1-SD increase in the row variable (or for categorical variables, the percentage difference in mean values of the HGI for the category vs the reference).

^d^*P* < .001.

^e^*P* < .01.

^f^*P* < .05.

### HGI AND RISK OF CVD MORTALITY

**Figure 2.**
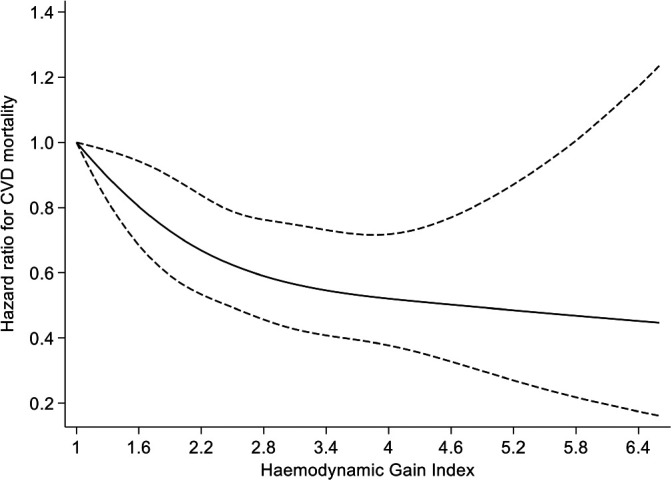
Restricted cubic splines of the HR of cardiovascular disease mortality with the hemodynamic gain index. Reference value for the hemodynamic gain index is 1.0; dashed lines represent 95% CI for the spline model (solid line). Models were adjusted for age, smoking status, history of type 2 diabetes, total cholesterol, high-density lipoprotein cholesterol, body mass index, fasting plasma glucose, alcohol consumption, prevalent coronary heart disease, use of cholesterol medication, prevalent atrial fibrillation, total physical activity, socioeconomic status, and high-sensitivity C-reactive protein. Abbreviation: CVD, cardiovascular disease.

**Figure 3.**
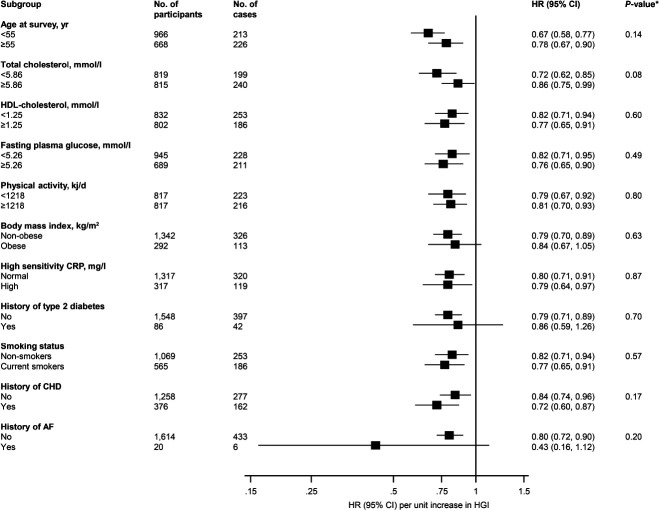
Association of the hemodynamic gain index with cardiovascular disease mortality in clinically relevant subgroups. HRs were adjusted for age, smoking status, history of type 2 diabetes, total cholesterol, high-density lipoprotein cholesterol, body mass index, fasting plasma glucose, alcohol consumption, prevalent coronary heart disease, use of cholesterol medication, prevalent atrial fibrillation, total physical activity, socioeconomic status, and high-sensitivity C-reactive protein. Abbreviations: AF, atrial fibrillation; CHD, coronary heart disease; CRP, C-reactive protein; HDL, high-density lipoprotein; HGI, hemodynamic gain index. *P value for interaction; cut-offs used for age, total cholesterol, high-density lipoprotein cholesterol, fasting plasma glucose, and physical activity are median values; nonobese and obese were defined as body mass index <30 and ≥30 kg/m^2^, respectively; normal and high CRP levels were defined as ≤3.0 and >3 mg/L, respectively.

A total of 439 CVD mortality cases were recorded during a median (IQR) follow-up of 28.7 (19.0, 31.4) yr (40 058 person-yr at risk). A multivariable restricted cubic spline curve showed the risk of CVD mortality decreased continuously with the increasing HGI from to 1.1-5.7 bpm/mm Hg (*P* value for nonlinearity = .28), beyond which there was no significant decrease in CVD mortality risk (Figure 2). In an analysis adjusted for age, smoking status, history of T2D, total cholesterol, HDL-C, BMI, FPG, alcohol consumption, prevalent CHD, use of cholesterol medication, prevalent AF, total PA, SES, and hsCRP, HR (95% CI) per 1 unit higher HGI for CVD mortality was 0.80 (0.71-0.89), which was attenuated to null on further adjustment for CRF 0.92 (0.81-1.04) (Table 2). Alternatively, comparing the top versus bottom tertiles of the HGI, the corresponding adjusted HRs (95% CI) for CVD mortality were 0.65 (0.50-0.86) and 0.88 (0.65-1.19), respectively. The association did not significantly differ according to the levels or categories of relevant clinical characteristics (Figure 3). Given the high mortality rate during follow-up (988 events), we assessed the association between the HGI and all-cause mortality risk in subsidiary analysis. The associations were similar to that between the HGI and CVD mortality risk (see Supplemental Digital Content 2, available at: http://links.lww.com/JCRP/A454).

**Table 2:** Association Between the Hemodynamic Gain Index and Risk of Cardiovascular Mortality

		Model 1^a^		Model 2^b^		Model 3^c^	
HGI, bpm/mm Hg	Events/Total	HR (95% CI)	*P* Value	HR (95% CI)	*P* Value	HR (95% CI)	*P* Value
Per unit increase	439/1634	0.64 (0.58-0.71)	<.001	0.80 (0.71-0.89)	<.001	0.92 (0.81-1.04)	.20
Tertile 1 (<2.07)	210/545	Reference		Reference		Reference	
Tertile 2 (2.08-2.94)	140/545	0.58 (0.46-0.71)	<.001	0.81 (0.65-1.02)	.073	0.98 (0.77-1.25)	.87
Tertile 3 (>2.94)	89/544	0.38 (0.30-0.50)	<.001	0.65 (0.50-0.86)	.003	0.88 (0.65-1.19)	.42

Abbreviation: HGI, hemodynamic gain index.

^a^Model 1: Adjusted for age.

^b^Model 2: Model 1 plus smoking status, history of type 2 diabetes, total cholesterol, high-density lipoprotein cholesterol, body mass index, fasting plasma glucose, alcohol consumption, prevalent coronary heart disease, use of cholesterol medication, prevalent atrial fibrillation, total physical activity, socioeconomic status, and high-sensitivity C-reactive protein.

^c^Model 3: Model 2 plus cardiorespiratory fitness.

In another analysis that assessed the association of CRF with CVD mortality risk in the same set of study participants with consistent adjustment for confounders, the significant association remained following further adjustment for the HGI (Table 3).

**Table 3:** Association Between Cardiorespiratory Fitness and Risk of Cardiovascular Mortality

		Model 1^a^		Model 2^b^		Model 3^c^	
CRF, METs	Events/Total	HR (95% CI)	*P* Value	HR (95% CI)	*P* Value	HR (95% CI)	*P* Value
Per unit increase	439/1634	0.75 (0.72-0.79)	<.001	0.84 (0.79-0.89)	<.001	0.86 (0.80-0.92)	<.001
Tertile 1 (2.11-7.73)	235/545	Reference		Reference		Reference	
Tertile 2 (7.74-9.61)	130/545	0.47 (0.38-0.59)	<.001	0.62 (0.49-0.78)	<.001	0.66 (0.52-0.85)	.001
Tertile 3 (>9.61)	74/544	0.26 (0.20-0.34)	<.001	0.45 (0.33-0.61)	<.001	0.50 (0.36-0.70)	<.001

Abbreviations: CRF, cardiorespiratory fitness; METs, metabolic equivalents.

^a^Model 1: Adjusted for age.

^b^Model 2: Model 1 plus smoking status, history of type 2 diabetes, total cholesterol, high-density lipoprotein cholesterol, body mass index, fasting plasma glucose, alcohol consumption, prevalent coronary heart disease, use of cholesterol medication, prevalent atrial fibrillation, total physical activity, socioeconomic status, and high-sensitivity C-reactive protein.

^c^Model 3: Model 2 plus the hemodynamic gain index.

### CVD MORTALITY RISK PREDICTION

A CVD mortality risk prediction model containing traditional risk factors yielded a C-index of 0.6998 (95% CI, 0.6720-0.7275). After addition of information on the HGI, the C-index was 0.7283 (95% CI, 0.7017-0.7549), representing a significant increase of 0.0285 (95% CI, 0.0130-0.0440; *P* < .001). The −2 log likelihood was significantly improved on addition of the HGI to the risk model (*P* for comparison < .001). The net reclassification improvement and integrated discrimination improvement were 8.34% (95% CI, 3.30-13.38; *P* < .001) and 0.0291 (95% CI, 0.0201-0.0382; *P* < .001), respectively (see Supplemental Digital Content 3, available at: http://links.lww.com/JCRP/A455).

There was a C-index change of 0.0413 (95% CI, 0.0226-0.0599; *P* = .001), after adding CRF to the model containing traditional risk factors. The −2 log likelihood was significantly improved on addition of absolute CRF to the model (*P* for comparison < .001). There was a significant improvement in the classification of participants into CVD mortality risk categories (net reclassification improvement, 14.74%, 8.62-20.86; *P* = .01). The integrated discrimination improvement was 0.0516 (0.0394-0.0638; *P* < .001) (see Supplemental Digital Content 3. available at: http://links.lww.com/JCRP/A455). The improvement in the C-index provided by CRF was 0.0128 higher (95% CI, −0.0015 to 0.0270; *P* = .08) than that of the HGI.

## DISCUSSION

In a general population of middle-aged and older men, the higher HGI during CPX was associated with a lower risk of CVD mortality in a graded fashion. The association was independent of several CVD risk factors and potential confounders, but it was partly dependent on CRF levels. Similar associations were observed between the HGI and all-cause mortality risk, which was not surprising because CVD was the most common cause of death in the cohort. The association between the HGI and CVD mortality risk was not modified by relevant clinical characteristics including age, BMI, FPG, and comorbidities such as T2D, CHD, and AF. Addition of information on the HGI to a CVD mortality risk prediction model containing traditional risk factors showed improvements in risk discrimination and reclassification across clinical risk categories at 25 yr. Comparison analyses in the same set of participants suggested that CRF was a stronger risk indicator and predictor for CVD mortality than the HGI.

This is the first study to evaluate the prospective association between the HGI derived from exercise testing and the risk of CVD mortality, as well as the potential utility of the HGI in long-term CVD mortality risk prediction. The HGI is a novel index that was developed from HR and BP responses during CPX; the first seminal study conducted in men was published in 2019.^17^ Given that it is well documented that hemodynamic responses (HR and BP) to exercise testing are independently associated with CVD and are also important determinants of prognosis,^13^,^15^,^16^ Vainshelboim and colleagues^17^ sought to develop a simple but useful noninvasive hemodynamic marker from these responses, which could be used to optimize hemodynamic assessment and enhance risk stratification. Findings from their validation studies conducted separately in men and women showed that the higher HGI was independently associated with a decreased all-cause mortality risk, and remained robust in several sensitivity analyses.^17^,^19^ Chaikijurajai and colleagues^18^ have also recently shown that the lower HGI was independently associated with an increased all-cause mortality risk, and this association persisted in several clinically relevant subgroups including men and women.

Heart rate and SBP responses to maximal exercise testing are used in deriving the HGI. It is well established that abnormal HR and SBP at rest and during exercise are related to an increased risk of adverse CVD outcomes including mortality.^13^,^16^ The product of HR and SBP during exercise represents the rate-pressure product, which is considered an indirect measure of myocardial oxygen uptake and cardiac function^45^ and has also been shown to be related to cardiac function and prognosis in patients with coronary artery disease.^45^ The HGI reflects the pumping capacity of the left ventricle and the compliance of the vasculature in response to demanding physiological effort during maximal aerobic exercise and is a good measure of the ability of the cardiovascular system to generate blood flow forcefully to meet the physiological demands of the body.^17^ The higher the HGI during exercise, the more it is reflective of good cardiac function, and protective for mortality.^17^

It is well documented that CPX parameters such as CRF as measured by V˙o_2peak_ or V˙o_2max_, V˙o_2_ at aerobic or ventilatory threshold, and the cardiorespiratory optimal point are independently associated with cardiovascular outcomes and provide diagnostic and prognostic information for general population participants and specific patient populations.^15^,^26^,^27^,^46^ It is also well established that HR and SBP responses during exercise testing are useful for the diagnosis of CVD and for determining overall prognosis.^13^,^15^,^16^ Though our results suggest that CRF is a better risk indicator and predictor than the HGI, the HGI is a simple, noninvasive, well-validated, and sensitive hemodynamic marker that combines HR and BP responses into one metric and has potential value for long-term risk stratification of CVD mortality in clinical settings. Our findings extend those of Vainshelboim and colleagues,^17^,^19^ which suggest that the HGI may be a powerful prognostic marker for all-cause mortality in men and women. Further studies are needed in other general population and specific patient settings to evaluate the potential clinical application of the HGI.

### STRENGTHS AND LIMITATIONS

This is the first study on the association between the HGI and the risk of CVD mortality. We employed a relatively large ethnically and genetically homogeneous nationally representative sample that was prospectively followed over two decades. Our analysis was comprehensive and included detailed evaluation of the nature, magnitude, and specificity of the association—adjustment for several relevant covariates, assessment of the dose-response relationship, and subgroup analyses in clinically relevant groups. We also evaluated the prognostic ability of the HGI beyond established risk factors using measures of discrimination and reclassification. Finally, we compared the HGI with CRF as risk and prognostic indicators for CVD mortality in the same sample of participants. However, the assessment of the HGI based on HR and SBP data does not necessarily need to respiratory gas exchange analysis during CPX. The following limitations deserve consideration: (i) the findings cannot be generalized to women, other age groups, and other populations, especially given the racial differences in CRF and BP responses to exercise^47^,^48^; (ii) the HGI was assessed using single baseline values of HR and SBP, hence, the potential for regression dilution bias due to the long-term follow-up of the cohort; (iii) use of medication during the follow-up was not available; and (iv) due to the observational design, there was potential for reverse causation and residual confounding due to errors in measured variables and relevant unmeasured confounders.

## CONCLUSIONS

The higher HGI during CPX is associated with a lower risk of CVD mortality in a graded fashion, but it is partly dependent on CRF. Also, the HGI significantly improves the prediction and classification of the long-term risk for CVD mortality beyond established risk factors.

## Supplementary Material

## ACKNOWLEDGMENTS

This work was supported by the Finnish Foundation for Cardiovascular Research, Helsinki, Finland. The funders had no role in study design, data collection and analysis, decision to publish, or preparation of the manuscript. We thank the staff of the Kuopio Research Institute of Exercise Medicine and the Research Institute of Public Health and University of Eastern Finland, Kuopio, Finland, for the data collection in the study.

## References

[1] World Health Organization. Fact sheets. The top 10 causes of death. https://www.who.int/news-room/fact-sheets/detail/the-top-10-causes-of-death. Accessed Sep 10, 2021.

[2] Wood D. Established and emerging cardiovascular risk factors. Am Heart J. 2001;141(2 suppl):S49–S57. 11174359 10.1067/mhj.2001.109951

[3] Ross R Blair SN Arena R Importance of assessing cardiorespiratory fitness in clinical practice: a case for fitness as a clinical vital sign: a scientific statement from the American Heart Association. Circulation. 2016;134(24):e653–e699. 27881567 10.1161/CIR.0000000000000461

[4] Kodama S Saito K Tanaka S Cardiorespiratory fitness as a quantitative predictor of all-cause mortality and cardiovascular events in healthy men and women: a meta-analysis. JAMA. 2009;301(19):2024–2035. 19454641 10.1001/jama.2009.681

[5] Hagnas MJ Kurl S Rauramaa R The value of cardiorespiratory fitness and exercise-induced ST segment depression in predicting death from coronary heart disease. Int J Cardiol. 2015;196:31–33. 26070181 10.1016/j.ijcard.2015.05.134

[6] Laukkanen JA Makikallio TH Rauramaa R Kiviniemi V Ronkainen K Kurl S. Cardiorespiratory fitness is related to the risk of sudden cardiac death: a population-based follow-up study. J Am Coll Cardiol. 2010;56(18):1476–1483. 20951323 10.1016/j.jacc.2010.05.043

[7] Letnes JM Dalen H Vesterbekkmo EK Wisloff U Nes BM. Peak oxygen uptake and incident coronary heart disease in a healthy population: the HUNT Fitness Study. Eur Heart J. 2019;40(20):1633–1639. 30496487 10.1093/eurheartj/ehy708

[8] Imboden MT Harber MP Whaley MH The association between the change in directly measured cardiorespiratory fitness across time and mortality risk. Prog Cardiovasc Dis. 2019;62(2):157–162. 30543812 10.1016/j.pcad.2018.12.003

[9] Laukkanen JA Isiozor NM Kunutsor SK. Objectively assessed cardiorespiratory fitness and all-cause mortality risk: an updated meta-analysis of 37 cohort studies involving 2,258,029 participants. Mayo Clin Proc. 2022;97(6):1054–1073. 35562197 10.1016/j.mayocp.2022.02.029

[10] Laukkanen JA Kunutsor SK Yates T Prognostic relevance of cardiorespiratory fitness as assessed by submaximal exercise testing for all-cause mortality: a UK Biobank prospective study. Mayo Clin Proc. 2020;95(5):867–878. 32370851 10.1016/j.mayocp.2019.12.030

[11] Laukkanen JA Kurl S Khan H Zaccardi F Kunutsor SK. Percentage of age-predicted cardiorespiratory fitness is inversely associated with cardiovascular disease mortality: a prospective cohort study. Cardiology. 2021;146(5):616–623. 34198307 10.1159/000516123

[12] Myers J Prakash M Froelicher V Do D Partington S Atwood JE. Exercise capacity and mortality among men referred for exercise testing. N Engl J Med. 2002;346(11):793–801. 11893790 10.1056/NEJMoa011858

[13] Balady GJ Arena R Sietsema K Clinician's guide to cardiopulmonary exercise testing in adults: a scientific statement from the American Heart Association. Circulation. 2010;122(2):191–225. 20585013 10.1161/CIR.0b013e3181e52e69

[14] Lavie CJ Arena R Swift DL Exercise and the cardiovascular system: clinical science and cardiovascular outcomes. Circ Res. 2015;117(2):207–219. 26139859 10.1161/CIRCRESAHA.117.305205 PMC4493772

[15] Guazzi M Arena R Halle M Piepoli MF Myers J Lavie CJ. 2016 focused update: clinical recommendations for cardiopulmonary exercise testing data assessment in specific patient populations. Eur Heart J. 2018;39(14):1144–1161. 27141094 10.1093/eurheartj/ehw180

[16] Guazzi M Adams V Conraads V EACPR/AHA scientific statement. Clinical recommendations for cardiopulmonary exercise testing data assessment in specific patient populations. Circulation. 2012;126(18):2261–2274. 22952317 10.1161/CIR.0b013e31826fb946 PMC4777325

[17] Vainshelboim B Kokkinos P Myers J. Prognostic Value and clinical usefulness of the hemodynamic gain index in men. Am J Cardiol. 2019;124(4):644–649. 31196561 10.1016/j.amjcard.2019.05.015

[18] Chaikijurajai T Wu Y Grodin JL Harb S Jaber W Tang WHW. Prognostic value of the hemodynamic gain index in different groups of patients undergoing cardiopulmonary exercise stress testing. Eur Heart J. 2021;42(suppl 1):ehab724.2672. 10.1016/j.ahjo.2022.100174 PMC9354505 35935015

[19] Vainshelboim B Kokkinos P Myers J. Hemodynamic gain index in women: a validation study. Int J Cardiol. 2020;308:15–19. 32248965 10.1016/j.ijcard.2020.03.066

[20] Chaikijurajai T Wu Y Engelman T Clinical significance of the hemodynamic gain index in patients with heart failure with reduced ejection fraction undergoing cardiopulmonary exercise testing. J Amer Col Cardiol. 2021;77(18, suppl 1):810–810.

[21] von Elm E Altman DG Egger M Pocock SJ Gotzsche PC Vandenbroucke JP. The Strengthening the Reporting of Observational Studies in Epidemiology (STROBE) statement: guidelines for reporting observational studies. J Clin Epidemiol. 2008;61(4):344–349. 18313558 10.1016/j.jclinepi.2007.11.008

[22] Laukkanen JA Lavie CJ Khan H Kurl S Kunutsor SK. Cardiorespiratory fitness and the risk of serious ventricular arrhythmias: a prospective cohort study. Mayo Clin Proc. 2019;94(5):833–841. 30935710 10.1016/j.mayocp.2018.11.027

[23] Laukkanen JA Kurl S Rauramaa R Lakka TA Venalainen JM Salonen JT. Systolic blood pressure response to exercise testing is related to the risk of acute myocardial infarction in middle-aged men. Eur J Cardiovasc Prev Rehabil. 2006;13(3):421–428. 16926673 10.1097/01.hjr.0000198915.83234.59

[24] Savonen KP Kiviniemi V Laukkanen JA Chronotropic incompetence and mortality in middle-aged men with known or suspected coronary heart disease. Eur Heart J. 2008;29(15):1896–1902. 18556711 10.1093/eurheartj/ehn269

[25] Kunutsor SK Khan H Laukkanen JA. Serum albumin concentration and incident type 2 diabetes risk: new findings from a population-based cohort study. Diabetologia. 2015;58(5):961–967. 25680582 10.1007/s00125-015-3520-0

[26] Kunutsor SK Kurl S Khan H Zaccardi F Laukkanen JA. Associations of cardiovascular and all-cause mortality events with oxygen uptake at ventilatory threshold. Int J Cardiol. 2017;236:444–450. 28209387 10.1016/j.ijcard.2017.01.156

[27] Kunutsor SK Kurl S Khan H Zaccardi F Rauramaa R Laukkanen JA. Oxygen uptake at aerobic threshold is inversely associated with fatal cardiovascular and all-cause mortality events. Ann Med. 2017;49(8):698–709. 28805463 10.1080/07853890.2017.1367958

[28] Kunutsor SK Laukkanen JA Kauhanen J Willeit P. Physical activity may not be associated with long-term risk of dementia and Alzheimer's disease. Eur J Clin Invest. 2021;51(3):e13415. 32991743 10.1111/eci.13415 PMC7988584

[29] Kunutsor SK Jae SY Makikallio TH Laukkanen JA. High fitness levels attenuate the increased risk of heart failure due to low socioeconomic status: a cohort study. Eur J Clin Invest. 2022;52(6):e13744. 35032034 10.1111/eci.13744 PMC9285703

[30] Kunutsor SK Jae SY Laukkanen JA. Impact of sauna bathing on risk of pneumonia in men with low socioeconomic status: a cohort study. J Cardiopulm Rehabil Prev. 2021;41(4):289–291. 34158458 10.1097/HCR.0000000000000611

[31] Kunutsor SK Jae SY Makikallio TH Laukkanen JA. High fitness levels attenuate the increased risk of hypertension due to low socioeconomic status in middle-aged men: a cohort study. J Cardiopulm Rehabil Prev. 2022;42(2):134–136. 35121703 10.1097/HCR.0000000000000673

[32] Karppi J Kurl S Makikallio TH Ronkainen K Laukkanen JA. Serum beta-carotene concentrations and the risk of congestive heart failure in men: a population-based study. Int J Cardiol. 2013;168(3):1841–1846. 23333366 10.1016/j.ijcard.2012.12.072

[33] Therneau TM Grambsch PM. Modeling Survival Data: Extending the Cox Model. New York, NY: Springer; 2000.

[34] Harrell FE Jr. Regression Modeling Strategies: With Applications to Linear Models, Logistic Regression, and Survival Analysis. New York, NY: Springer; 2001.

[35] Laukkanen T Kunutsor SK Khan H Willeit P Zaccardi F Laukkanen JA. Sauna bathing is associated with reduced cardiovascular mortality and improves risk prediction in men and women: a prospective cohort study. BMC Med. 2018;16(1):219. 30486813 10.1186/s12916-018-1198-0 PMC6262976

[36] Kunutsor SK Khan H Laukkanen T Laukkanen JA. Joint associations of sauna bathing and cardiorespiratory fitness on cardiovascular and all-cause mortality risk: a long-term prospective cohort study. Ann Med. 2018;50(2):139–146. 28972808 10.1080/07853890.2017.1387927

[37] Harrell FE Jr Lee KL Mark DB. Multivariable prognostic models: issues in developing models, evaluating assumptions and adequacy, and measuring and reducing errors. Stat Med. 1996;15(4):361–387. 8668867 10.1002/(SICI)1097-0258(19960229)15:4<361::AID-SIM168>3.0.CO;2-4

[38] Pencina MJ D'Agostino RB Sr D'Agostino RB Jr, Vasan RS. Evaluating the added predictive ability of a new marker: from area under the ROC curve to reclassification and beyond. Stat Med. 2008;27(2):157–172; discussion 207-212. 17569110 10.1002/sim.2929

[39] Pencina MJ D'Agostino RB Sr Steyerberg EW. Extensions of net reclassification improvement calculations to measure usefulness of new biomarkers. Stat Med. 2011;30(1):11–21. 21204120 10.1002/sim.4085 PMC3341973

[40] Cook NR. Use and misuse of the receiver operating characteristic curve in risk prediction. Circulation. 2007;115(7):928–935. 17309939 10.1161/CIRCULATIONAHA.106.672402

[41] Harrell FEJ. Regression Modeling Strategies. New York, NY: Springer; 2001.

[42] Gupta S Rohatgi A Ayers CR Cardiorespiratory fitness and classification of risk of cardiovascular disease mortality. Circulation. 2011;123(13):1377–1383. 21422392 10.1161/CIRCULATIONAHA.110.003236 PMC3926656

[43] Laukkanen JA Kurl S Salonen R Rauramaa R Salonen JT. The predictive value of cardiorespiratory fitness for cardiovascular events in men with various risk profiles: a prospective population-based cohort study. Eur Heart J. 2004;25(16):1428–1437. 15321701 10.1016/j.ehj.2004.06.013

[44] Harber MP Kaminsky LA Arena R Impact of cardiorespiratory fitness on all-cause and disease-specific mortality: advances since 2009. Prog Cardiovasc Dis. 2017;60(1):11–20. 28286137 10.1016/j.pcad.2017.03.001

[45] Fletcher GF Ades PA Kligfield P Exercise standards for testing and training: a scientific statement from the American Heart Association. Circulation. 2013;128(8):873–934. 23877260 10.1161/CIR.0b013e31829b5b44

[46] Peterman JE Harber MP Fleenor BS Whaley MH Araujo CG Kaminsky LA. Cardiorespiratory optimal point is a submaximal exercise test variable and a predictor of mortality risk: the Ball State Adult Fitness Longitudinal Lifestyle Study (BALL ST). J Cardiopulm Rehabil Prev. 2022;42(6):E90–E96. 35861956 10.1097/HCR.0000000000000711 PMC9662820

[47] Sabbahi A Arena R Kaminsky LA Characterization of the blood pressure response during cycle ergometer cardiopulmonary exercise testing in black and white men: Data from the Fitness Registry and Importance of Exercise: a National Database (FRIEND). J Hum Hypertens. 2021;35(8):685–695. 32868881 10.1038/s41371-020-00411-5 PMC8900149

[48] Canada JM Park TS Ravindra K Comparison of cardiorespiratory fitness in Black or African American versus Caucasian patients with heart failure. J Cardiopulm Rehabil Prev. 2022;42(1):39–44. 34793367 10.1097/HCR.0000000000000605 PMC8602869

