# Supplementary material for: Hemodynamic Gain Index Is Associated With Cardiovascular Mortality and Improves Risk Prediction: A PROSPECTIVE COHORT STUDY
Source: J Cardiopulm Rehabil Prev. 2023 Mar 6;43(5):368–76. doi: 10.1097/HCR.0000000000000777 (PMC10467812; doi:10.1097/HCR.0000000000000777)
Supplement: Supplementary file 2 [file jcprh-43-368-s002.docx]

**SDC 2.** Association between hemodynamic gain index and all-cause mortality risk

| **HGI, bpm/mm Hg** | **Events/**  **Total** | **Model 1** |  | **Model 2** |  | **Model 3** |  |
| --- | --- | --- | --- | --- | --- | --- | --- |
|  |  | HR (95% CI) | *P-*value | HR (95% CI) | *P*-value | HR (95% CI) | *P*-value |
| Per unit increase | 988 / 1634 | 0.73 (0.68-0.78) | <.001 | 0.82 (0.76-0.89) | <.001 | 0.92 (0.84-1.00) | .039 |
| Tertile 1 (<2.07) | 415 / 545 | ref |  | ref |  | ref |  |
| Tertile 2 (2.08-2.94) | 341 / 545 | 0.70 (0.61-0.81) | <.001 | 0.86 (0.74-1.00) | .048 | 0.98 (0.84-1.15) | .84 |
| Tertile 3 (>2.94) | 232 / 544 | 0.49 (0.42-0.58) | <.001 | 0.69 (0.57-0.82) | <.001 | 0.87 (0.71-1.05) | .15 |

Abbreviations: CRF, cardiorespiratory fitness; HGI, hemodynamic gain index; ref, reference

Model 1: Adjusted for age

Model 2: Model 1 plus smoking status, history of type 2 diabetes, total cholesterol, and high-density lipoprotein cholesterol, body mass index, fasting plasma glucose, alcohol consumption, prevalent coronary heart disease, use of cholesterol medication, prevalent atrial fibrillation, total physical activity, and socioeconomic status, and high-sensitivity C-reactive protein

Model 3: Model 2 plus CRF
