# Supplementary material for: Hemodynamic Gain Index Is Associated With Cardiovascular Mortality and Improves Risk Prediction: A PROSPECTIVE COHORT STUDY
Source: J Cardiopulm Rehabil Prev. 2023 Mar 6;43(5):368–76. doi: 10.1097/HCR.0000000000000777 (PMC10467812; doi:10.1097/HCR.0000000000000777)
Supplement: Supplementary file 3 [file jcprh-43-368-s003.docx]

**SDC 3.** Risk discrimination and reclassification upon addition of HGI and CRF to a CVD mortality risk prediction model containing conventional risk factors

| **Discrimination** | **HGI** | **CRF** |
| --- | --- | --- |
| C-index (95% CI): conventional risk factors | 0.6998 (0.6720, 0.7275) | 0.6998 (0.6720, 0.7275) |
| C-index (95% CI): conventional risk factors plus exposure | 0.7283 (0.7017, 0.7549) | 0.7411 (0.7152, 0.7670) |
| C-index change (95% CI) | 0.0285 (0.0130, 0.0440) | 0.0413 (0.0226, 0.0599) |
| *P*-value | <.001 | .001 |
| *P*-value for difference in -2 log likelihood | <.001 | <.001 |
|  |  |  |
| **Reclassification** |  |  |
| Net reclassification index (95% CI) | 8.34% (3.30, 13.38) | 14.74% (8.62, 20.86) |
| *P* -value | <.001 | .01 |
|  |  |  |
| Integrated discrimination index (95% CI) | 0.0291 (0.0201, 0.0382) | 0.0516 (0.0394, 0.0638) |
| *P* -value | <.001 | <.001 |

The model with conventional risk factors included age, history of type 2 diabetes, total cholesterol, high-density lipoprotein cholesterol and smoking.

Abbreviations: CRF, cardiorespiratory fitness; CVD, cardiovascular disease; HGI, hemodynamic gain index
